# Corticosteroid effects on ventilator-induced diaphragm dysfunction in anesthetized rats depend on the dose administered

**DOI:** 10.1186/1465-9921-11-178

**Published:** 2010-12-14

**Authors:** Karen Maes, Anouk Agten, Ashley Smuder, Scott K Powers, Marc Decramer, Ghislaine Gayan-Ramirez

**Affiliations:** 1 Respiratory Muscle Research Unit, Laboratory of Pneumology and Respiratory Division, Katholieke Universiteit Leuven, B-3000 Leuven Belgium; 2 Department of Applied Physiology and Kinesiology, University of Florida, Gainesville, Florida, USA

## Abstract

**Background:**

High dose of corticosteroids has been previously shown to protect against controlled mechanical ventilation (CMV)-induced diaphragmatic dysfunction while inhibiting calpain activation. Because literature suggests that the calpain inhibiting effect of corticosteroid depends on the dose administered, we determined whether lower doses of corticosteroids would also provide protection of the diaphragm during CMV. This may be important for patients undergoing mechanical ventilation and receiving corticosteroids.

**Methods:**

Rats were assigned to controls or to 24 hours of CMV while being treated at the start of mechanical ventilation with a single intramuscular administration of either saline, or 5 mg/kg (low MP) or 30 mg/kg (high MP) of methylprednisolone.

**Results:**

Diaphragmatic force was decreased after CMV and this was exacerbated in the low MP group while high MP rescued this diaphragmatic dysfunction. Atrophy was more severe in the low MP group than after CMV while no atrophy was observed in the high MP group. A significant and similar increase in calpain activity was observed in both the low MP and CMV groups whereas the high dose prevented calpain activation. Expression of calpastatin, the endogenous inhibitor of calpain, was decreased in the CMV and low MP groups but its level was preserved to controls in the high MP group. Caspase-3 activity increased in all CMV groups but to a lesser extent in the low and high MP groups. The 20S proteasome activity was increased in CMV only.

**Conclusions:**

Administration of 30 mg/kg methylprednisolone during CMV protected against CMV-induced diaphragm dysfunction while 5 mg/kg was more deleterious. The protective effect is due mainly to an inhibition of the calpain system through preservation of calpastatin levels and to a lesser extent to a caspase-3 inhibition.

## Background

Corticosteroids are among the most widely used drugs in the world and are effective in the treatment of many inflammatory and immune diseases. However, one of the main side-effects of systemically administered corticosteroids is skeletal muscle myopathy, involving respiratory as well as peripheral muscles. The incidence of steroid-induced myopathy varies from 7% [1] to 60% [2] in patients receiving glucocorticoid treatment for various diseases. Glucocorticoids have been shown to cause mainly atrophy of fast-twitch type II muscle fibers with less or no impact on type I fibers [3]. In skeletal muscle, glucocorticoids decrease the rate of muscle protein synthesis and increase the rate of muscle proteolysis [4]. The stimulatory effect of corticosteroids on muscle proteolysis results from the activation of the proteolytic systems such as the ubiquitin-proteasome system (UPS), the lysosomal system, the calcium-dependent calpain system and the caspase-3 system [5,6].

Although the effects of corticosteroids on muscle proteolysis are well documented, the protective effect of corticosteroids on protein degradation is less recognized. In some circumstances, corticosteroids have been shown to inhibit the calpain system [7-10] and the caspase-3 system [11-13]. For calpain, *in vitro *degradation of neurofilament proteins from rat spinal cord homogenates through calpain activation, was substantially inhibited by corticosteroids in a dose-dependent fashion [7]. Also, in a rat model of ischemia-induced liver injury, pretreatment with prednisolone (10 mg/kg, corresponding ~1.6 mg/kg in humans) abolished calpain activation in the liver [14]. Interestingly, in this study the calpain-inhibiting effect of corticosteroids was shown to depend on the dose administered, being minimal at low concentrations. Recently our group showed that administration of a single high dose of methylprednisolone (80 mg/kg, corresponding ~13 mg/kg in humans) during controlled mechanical ventilation protected the diaphragm from the deleterious effects of prolonged mechanical ventilation through inhibition of the calpain system [9]. This study and a previous CMV study, in which we used a calpain inhibitor [9], confirm the important role of the calpain system in the development of VIDD. It is known that three major proteolytic systems are upregulated in the diaphragm during mechanical ventilation: the ubiquitin proteasome system (UPP), the Ca^2+^-dependent calpain system and the lysosomal system [15-17]. Although the UPP is considered a major proteolytic system in skeletal muscle, it cannot degrade intact myofilaments. Release of myofilaments for subsequent degradadtion by the UPP occurs by the calpain and/or caspase system and may be the rate-limiting step in skeletal muscle proteolysis[18].

In regard to patients undergoing prolonged mechanical ventilation, it is important to know whether lower doses of corticosteroids, as used in the clinical practice, can also provide protection against mechanical ventilation-induced diaphragmatic weakness.

Since the literature supports the fact that the calpain-inhibiting effect of corticosteroids depends on the dose administered, the aim of the present study was to determine whether administration of lower doses of corticosteroids would provide protection against ventilator-induced diaphragm dysfunction (VIDD).

## Methods

### Experimental procedure

Male Wistar rats were randomly assigned to one of four experimental groups: control (C, n = 9), 24 hours of mechanical ventilation receiving an intramuscular injection of saline (CMV, n = 9) or methylprednisolone (MP) at a low dose (5 mg/kg, MP-5, n = 7) or at high dose (30 mg/kg, MP-30, n = 6). The study was approved by the animal experiments committee of the Medical Faculty of the Katholieke Universiteit Leuven. Rats were tracheotomized and the jugular vein was cannulated for continuous infusion of Pentobarbital. A catheter was inserted into the carotid artery to permit continuous blood pressure measurements and the collection of blood to measure blood gases. Body temperature was continuously maintained at 37°C. Rats received an intramuscular injection of either saline or methylprednisolone (5 mg/kg, corresponding ~0.8 mg/kg in humans [19]) or 30 mg/kg, (corresponding ~4.8 mg/kg in humans [19]) at the start of the 24 h mechanical ventilation protocol. The doses of methylprednisolone were pharmacologically scaled to the animal's metabolic rate which makes the dose compatible with human dosages. Appropriate conversion of drug doses from animal to humans can be calculated as previously recommended [19]. Upon completion of mechanical ventilation, the diaphragm was quickly excised and a strip was used for *in vitro *contractile properties, as described previously [20], while the remaining part was frozen for further analysis.

### Histochemistry

Serial sections of the costal diaphragm were stained with hematoxylin and eosin and for myofibrillar adenosine triphosphatase to determine cross-sectional area (CSA) and proportion of the fibers, as described previously[20].

### Western blot

Talin, αII-spectrin and calpastatin, the endogenous inhibitor of calpain I and II, were measured by western blotting. Proteolysis of talin, a preferential intracellular substrate of calpain, was investigated as an indirect measurement of calpain activity. Measurement of the caspase-3 mediated cleavage of αII-spectrin was used to assess caspase-3 activity. Diaphragm was homogenized in a buffer containing 100 mM KPO_4 _and total protein concentration was determined with the Bradford method. Proteins were separated on a polyacrylamide gel and transferred onto a polyvinyldifluoride membrane. Blots were incubated overnight at 4°C with a primary antibody against talin (Sigma), calpastatin (Sigma) or αII-spectrin (Tebu-Bio) and with the appropriate secondary antibodies. For calpastatin, data were corrected for alpha-tubulin (Sigma) to ensure equal loading. Since calpain activity and caspase-3 activity are expressed as the ratio between breakdown products and intact protein, corrections for equal loading with alpha-tubulin were not performed. Ponceau S staining was performed for each blot to ensure proper transfer of the proteins. Proteins were visualized with ECL (Amersham) and analyzed with the software package (Bio 1D) of the imaging system (Photo print, Vilber, France).

### 20S proteasome activity

To determine the impact of our experimental treatments on proteasome activation in the diaphragm, we used a well-established kinetic fluorometric assay [21].

### Statistical analysis

Statistical analysis was performed with the GraphPad prism software (version 4.01, GraphPad, San diego, CA, USA). Population distribution was evaluated with the D'Agostino and Pearson omnibus normality test. Comparisons between the groups were made by a one-way analysis of variance. When appropriate, a Newman-Keuls Multiple Comparison Test was performed post hoc. Correlation analyses were performed using Pearson's coefficient of correlation. Significance was established at p < 0.05. Values are reported as mean ± SD.

## Results

### Systemic and biologic response to CMV

Arterial blood pressure was similar between the 4 groups. Blood pH, PO_2 _and PCO_2 _were maintained within the normal levels and were not different between the groups (Table 1).

**Table 1:** Blood gas data and arterial blood pressure

	PaO_2 _(mmHg)	PaCO_2 _(mmHg)	pH	PA (mmHg)
CMV	148 ± 56	26 ± 4	7.57 ± 0.10	94 ± 29
MP-5	148 ± 59	34 ± 12	7.51 ± 0.12	95 ± 22
MP-30	121 ± 61	35 ± 10	7.45 ± 0.14	131 ± 31

Blood gases and arterial blood pressure in animals under controlled mechanical ventilation treated with either saline (CMV) or with 5 mg/kg methylprednisolone (MP-5) or 30 mg/kg of methylprednisolone (MP-30). For the control (C) group no hemodynamic measurements are available. Values are mean ± SD.

### Diaphragm *in vitro *contractile properties

**Figure 1:**
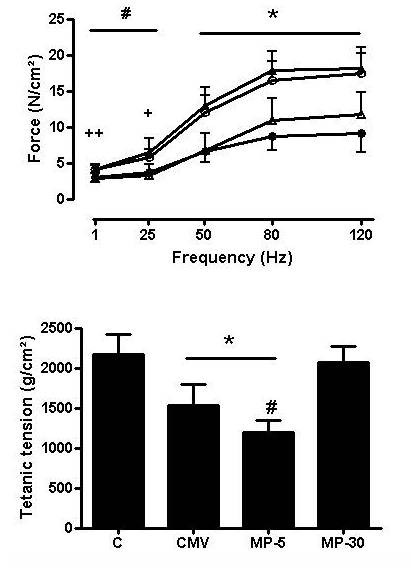
**Diaphragm force generation**. Upper panel: Force-frequency relationship in the diaphragm of controls (C, open rectangles, n = 9), controlled mechanical ventilation animals treated either with saline (CMV, closed triangles, n = 9) or with 5 mg/kg methylprednisolone (MP-5, open circles, n = 7) or 30 mg/kg methylprednisolone (MP-30, closed circles, n = 6). Maximal tetanic tension in the diaphragm of C, CMV, MP5 and MP30 groups. Values are means ± SD. *p < 0.001 CMV and MP-5 vs others, #p < 0.01 CMV vs C and MP-30, ++p < 0.01 MP-5 vs MP-30, +p < 0.05 MP-5 vs MP-30.

In the CMV group the force-frequency curve shifted downwards when compared to C, as previously shown (Figure 1 upper panel). In the MP-5 group, diaphragm force was further reduced compared to C and MP30. By contrast in the MP-30 group, diaphragm force was similar to that of C at all stimulation frequencies. Tetanic tension was decreased with 30% after CMV when compared to C and with an additional 15% in the MP-5 group (p < 0.01 vs others) while it was unchanged in the MP-30 group (Figure 1 lower panel).

### Histochemistry

**Figure 2:**
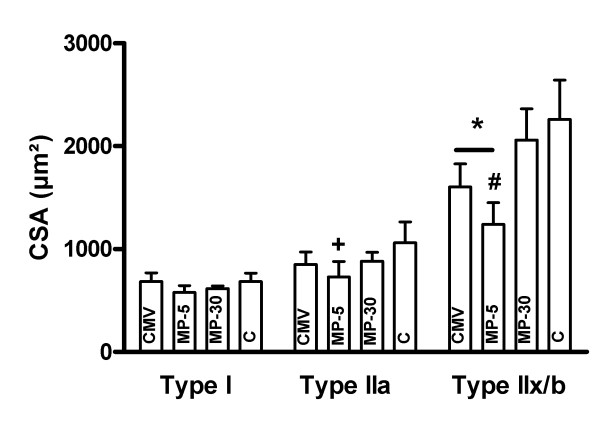
**Diaphragm cross-sectional area of the different fiber types**. Diaphragm cross-sectional area (CSA) of the type I, IIa and IIx/b fibers in the controls (C, n = 6) and controlled mechanical ventilation animals treated either with saline (CMV, n = 6) or with 5 mg/kg methylprednisolone (MP-5, n = 6) or 30 mg/kg methylprednisolone (MP-30, n = 5). Values are means ± SD. *p < 0.05 vs C and MP-30, #p < 0.05 vs CMV, +p < 0.01 vs C.

Proportions of the different fiber types were similar between all groups. Compared to C, diaphragm CSA of the type IIx/b fibers was significantly decreased with 29% after CMV, as previously shown, and with an additional 16% in the MP-5 group. CSA of the type IIa fibers were decreased in the MP-5 group only (-31% vs C, p < 0.01) (Figure 2). In the MP-30 group CSA of the different fiber types remained unchanged and similar to that of C.

### Western blot analysis of calpain, calpastatin and caspase-3

**Figure 3:**
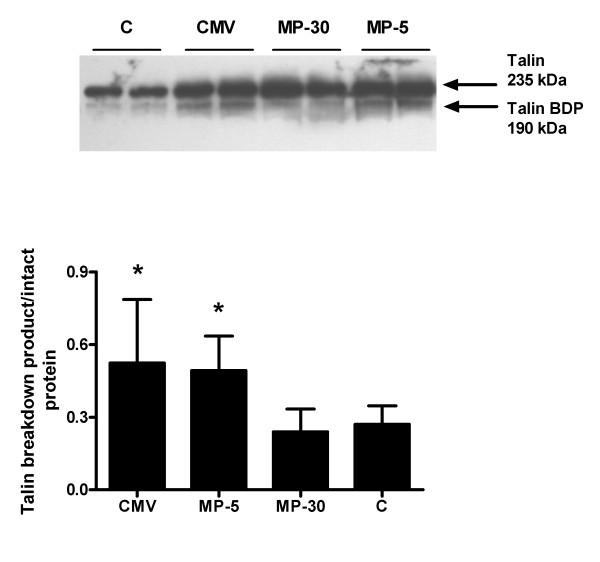
**Western blot analysis of talin**. Upper panel: A representative western blot for the analysis of intact and cleaved talin (BDP) in the diaphragm of controls (C, n = 6) and controlled mechanical ventilation with saline injection (CMV, n = 9) or with 5 mg/kg methylprednisolone (MP-5, n = 7) or 30 mg/kg methylprednisolone (MP-30, n = 5). Lower panel: Mean densitometric values for talin degradation by calpain. Values are shown as the ratio of degraded talin to intact protein and are expressed as means ± SD, *p < 0.05 vs others.

Calpain activity, measured by talin degradation, was significantly elevated after CMV, as previously shown, and to a similar extent in the MP-5 group when compared to C (Figure 3). In the MP-30 group, talin degradation was similar to control levels.

**Figure 4:**
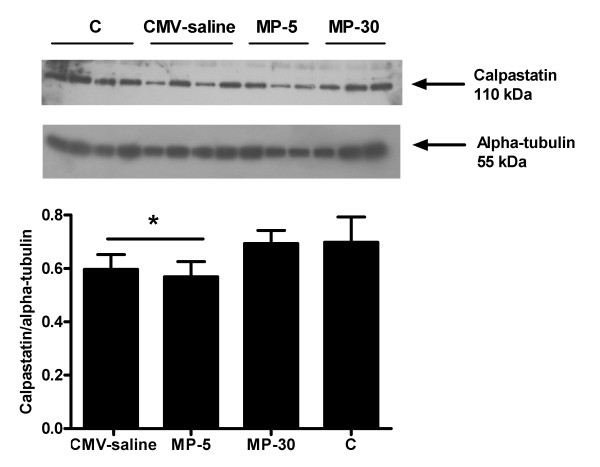
**Western blot analysis of calpastatin**. Upper panel: A representative western blot for calpastatin and α-tubulin in the diaphragm of controls (C, n = 6) and controlled mechanical ventilation with saline injection (CMV, n = 8) or with 5 mg/kg methylprednisolone (MP-5, n = 7) or 30 mg/kg methylprednsiolone (MP-30, n = 5). Lower panel: Densitometric values for calpastatin corrected for α-tubulin. Values are means ± SD, *p < 0.05 vs others.

Calpastatin levels were significantly and similarly decreased after CMV and after administration of 5 mg/kg MP compared with controls (Figure 4). In the MP-30 group, calpastatin expression was similar to that of the control group.

**Figure 5:**
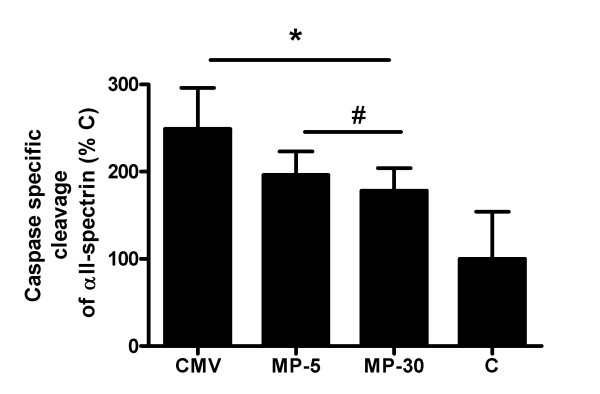
**Western blot analysis of αII-spectrin**. Densitometric values for αII-spectrin degradation by caspase-3. Values are shown as the ratio of degraded αII-spectrin to intact protein and are expressed as means ± SD, *p < 0.01 vs C, #p < 0.05 vs CMV.

Analysis of the caspase-3 mediated cleavage of αII-spectrin revealed that CMV induced a significant rise in caspase-3 activity when compared to C (+149%, p < 0.001). Caspase-3 activity was similarly increased in the MP-5 and the MP-30 group (+96% and +78% respectively, p < 0.05 vs C) but this increase was significantly less compared to that of CMV (Figure 5).

Significant negative correlations were found between calpain activity and diaphragm force (-0.50<r < -0.41, p < 0.05) as well as with CSA of the type IIx/b fibers (r = -0.57, p < 0.02). Significant positive correlation were observed between calpastatin and diaphragm force (0.43<r < 0.54, p < 0.05) and calpastatin and CSA of the type IIx/b fibers (r = 0.57, p < 0.02).

### 20S proteasome activity

Compared to control, the chymotrypsin-like activity of the 20S proteasome was increased (p < 0.05) by 48% in diaphragms from the CMV group. In contrast, both the low dose (MP-5) and high dose (MP-30) of corticosteroids prevented the CMV-induced proteasome activation in the diaphragm.

## Discussion

### Overview of principle findings

This is the first study to demonstrate that the protective effect of corticosteroids against VIDD depends on the dose administered and results mainly from an inhibition of the calpain system and to a lesser extent from a reduction in caspase-3 activation. Administration of relatively low dose of methylprednisolone (5 mg/kg) resulted in an exacerbation of diaphragm dysfunction and atrophy. None of these effects were observed with the higher dose of methylprednisolone (30 mg/kg), a dose that fully protected the diaphragm against the effects of CMV.

### Corticosteroids and skeletal muscle

Corticosteroids are known to decrease muscle synthesis and to accelerate protein degradation [4]. *In vivo *administration of corticosteroids to animals has been shown to stimulate the different proteolytic systems [22-26]. On the other hand, there are also evidences suggesting that corticosteroids may provide beneficial effects on skeletal muscles. In patients with Duchenne muscular dystrophy, treatment with prednisolone significantly improved muscle strength and this beneficial effect appeared to be associated with an increase in muscle mass probably mediated by inhibition of muscle proteolysis rather than by stimulation of muscle protein synthesis [27]. Inhibition of muscle proteolysis, in particular the calpain system, by corticosteroids has been suggested in several *in vitro *[7,19,28,29] and *in vivo *[14,30,31] studies. In addition, treatment with methylprednisolone has been shown to reduce caspase-3 mRNA and protein expression in several animal models [11-13].

### Corticosteroids and the calpain system

The ability of corticosteroids to inhibit calpain seems to depend on the dose administered. An *in vitro *study showed that methylprednisolone was slightly effective at low concentrations while more than 80% of calpain inhibition was observed with high concentrations[7]. This was also confirmed in several *in vivo *studies where different doses of corticosteroids were administered to animals. In rabbits, calpain activation caused by hypoxia was prevented by betametasone pretreatment, indicating inhibition of calpain activation [30]. In a rat model of ischemia-induced liver injury pretreatment of animals with 10 mg/kg of prednisolone (corresponding ~1.6 mg/kg in humans [19]) significantly inhibited calpain activation in the liver while lower doses (1 mg/kg, corresponding ~0.2 mg/kg in humans [19]and 3 mg/kg, corresponding ~0.5 mg/kg in humans [19]) did not [8]. Also a dose of 30 mg/kg of corticosteroids administered to piglets (corresponding ~6.5 mg/kg in humans [19]) before and during cardiopulmonary bypass was able to reduce the percentage of degraded troponin I while preserving calpastatin activity levels [31]. This is interesting knowing that the dose of 30 mg/kg is currently used in patients undergoing cardio-pulmonary bypass [32-34].

The precise mechanisms by which corticosteroids inhibit calpain activity remain unclear. Nonetheless, based upon our current knowledge regarding calpain regulation, a bride discussion of calpain regulation in the diaphragm during prolonged CMV is warranted. Calpain is a Ca^2+^-dependent cytosolic protease which is typically in an inactive state under basal conditions. Calcium is the most important activator of calpain. Binding of calcium to calpain leads to conformational changes of the molecule allowing activation of its catalytic site. An additional important regulator of calpain activity is its endogenous inhibitor calpastatin[18]. Evidence exists that corticosteroids might protect against calpain activation by preventing an increase in cytosolic calcium levels. In *mdx *muscle fibers, a condition in which cytosolic Ca^2+ ^is increased, treatment with methylprednisolone attenuated the rise in cytosolic free calcium following hypo-osmotic stress[35]. On the other hand, preservation of calpastatin levels was associated with calpain inhibition after MP treatment in a piglet model of cardiopulmonary bypass[31]. Therefore, in the current experiments, it is possible that the MP treatment inhibited CMV-induced calpain activation in the diaphragm by preventing an increase in cytosolic Ca^2+ ^levels, preservation of calpastatin levels, or some combination of both. Additional experiments will be required to provide a complete understanding of this issue.

### Corticosteroids and mechanical ventilation

Animal studies have clearly demonstrated that CMV impacts the diaphragm by promoting contractile dysfunction, increased proteolysis and atrophy [17,36]. Interestingly, our results reveal that administration of a relatively low dose of methylprednisolone (5 mg/kg, corresponding ~0.8 mg/kg in humans [19]) exacerbates the CMV-induced diaphragm dysfunction, whereas a higher dose (30 mg/kg, corresponding ~5 mg/kg in humans [19]) completely protected the diaphragm against VIDD. The dose-depending effect of corticosteroids are in agreement with previous studies [7,8,31]. Our finding of a negative correlation between calpain activity and either diaphragmatic force production or diaphragm fiber CSA further supports the notion that calpain activation plays an important role in CMV-induced diaphragmatic atrophy and contractile dysfunction. In our previous study we also showed that administration of 80 mg/kg (corresponding ~13 mg/kg in humans [19]) of MP during CMV protected against VIDD[9]. By contrast, CMV in combination with 80 mg/kg of MP in rabbits (corresponding ~28 mg/kg in humans [19]) showed no protection of VIDD after 1, 2 or 3 days of CMV [37]. It is unclear whether the discrepancy between our results and this work is related to species differences (rat vs rabbit) or to the duration of MP treatment. Furthermore, the present study also identified a potential role for calpastatin, the endogenous inhibitor of calpain, in the protective effect induced by corticosteroids during prolonged CMV. The positive correlation found in our study between calpastatin and diaphragm force or fiber dimensions further stress the potentially important role of calpastatin in this model. Inhibition of calpain by MP through a preservation of calpastatin levels has been previously reported in a model of cardiopulmonary bypass [31]. These findings coupled with our data suggest that high doses of corticosteroids may prevent loss of calpastatin and therefore prevent the activation of calpain in skeletal muscle. It is also possible that the way MP preserves diaphragm function during controlled mechanical ventilation might be related to intracellular cellular calcium levels. Indirect evidence suggests that prolonged CMV results in an increase in intracellular calcium levels in the diaphragm [15]. Therefore protection against CMV-induced increases in intracellular calcium levels and/or increasing calpastatin binding to calpain could be potential mechanisms by which corticosteroids prevent activation of the protease calpain and protect against VIDD. Both of these mechanisms have been proposed to explain the calpain inhibiting effect of prednisolone in the ischemic liver [14] and this protective effect of corticosteroids was shown to be dependent on the dose administered.

Surprisingly, our data showed a prevention of the CMV-induced increase in 20S proteasome activity with both doses of MP. This is in contrast with previous literature showing increases of several components of the ubiquitin proteasome system after corticosteroids treatment in *in vitro *[6,38] and in animal studies [22,39]. To our knowledge, only one *in vitro *study has demonstrated that treatment of cells (i.e., thymocytes) with dexamethasone decreased proteasome chymotrypsin-like activity in cell extracts [40]. Inhibition of the 20S proteasome activity, as observed in the present study, might be due to the fact that animals were treated with only a single injection of MP while in most other studies animals were treated repeatedly with corticosteroids.

Finally our data also indicate that caspase-3 activity was increased in the diaphragm after CMV and also, but to a lesser extent, after corticosteroids treatment independent of the dose used. Inhibition of caspase-3 by corticosteroids was previously shown in different animal models [11-13]. Indeed, administration of methylprednisolone (30 or 60 mg/kg) to piglets (corresponding ~6.5 or 13 mg/kg in humans [19]) with cardio-pulmonary bypass resulted in a reduction of myocardial caspase-3 activity [12]. Similar, when 10 mg/kg (corresponding ~1.6 mg/kg in humans [19]) of dexamethasone was administered to endotoxemic rats, the expression of caspase-3 mRNA in the brain was inhibited [13]. Currently, the mechanism of the inhibitory effect of steroids on caspase-3 expression remains unknown. In the present study, our data indicate that the effects of MP on caspase-3 activity during CMV were independent of the dose administered. Our data also clearly show that MP can minimize the deleterious effects of CMV on the diaphragm despite the fact that MP treatment did not fully prevent caspase-3 activation. We interpret these results as another indication of the main role played by the calpain system in the development of VIDD.

## Conclusions

The effects of corticosteroids on the diaphragm during CMV depend on the dose administered and relatively high doses of corticosteroids are required to provide protection against CMV-induced diaphragmatic atrophy and contractile dysfunction. The mechanism(s) responsible for high-dose glucocorticoid-induced diaphragmatic protection are uncertain but may be linked to the ability of high doses of corticosteroids to inhibit mainly calpain activity and caspase-3 but to a lesser extent. The effects on calpain activity may be related to calpastatin expression levels.

## Competing interests

The authors declare that they have no competing interests.

## Authors' contributions

KM participated in the study design, performed the experiments, data analysis and drafted the manuscript. AA helped to perform the experiments and to draft the manuscript. AS carried out the 20S proteasome activity measurement and analysis and helped drafting the manuscript. SP participated in the design of the study, interpretation of the data and helped drafting the manuscript. MD participated in the study design and conception and helped drafting the manuscript. GGR conceived the study, participated in the study design and coordination and helped to draft the manuscript. All authors read and approved the final manuscript.

## References

[B1] Askari A Vignos PJ Jr Moskowitz RW Steroid myopathy in connective tissue disease Am J Med 1976 61 485 492 10.1016/0002-9343(76)90327-2 973643

[B2] Batchelor TT Taylor LP Thaler HT Posner JB DeAngelis LM Steroid myopathy in cancer patients Neurology 1997 48 1234 1238 9153449 10.1212/wnl.48.5.1234

[B3] Dekhuijzen PN Gayan-Ramirez G Bisschop A de Bock V Dom R Bouillon R Rat diaphragm contractility and histopathology are affected differently by low dose treatment with methylprednisolone and deflazacort Eur Respir J 1995 8 824 830 7656957

[B4] Schakman O Gilson H Thissen JP Mechanisms of glucocorticoid-induced myopathy J Endocrinol 2008 197 1 10 10.1677/JOE-07-0606 18372227

[B5] Hasselgren PO Glucocorticoids and muscle catabolism Curr Opin Clin Nutr Metab Care 1999 2 201 205 10.1097/00075197-199905000-00002 10456248

[B6] Sacheck JM Ohtsuka A McLary SC Goldberg AL IGF-I stimulates muscle growth by suppressing protein breakdown and expression of atrophy-related ubiquitin ligases, atrogin-1 and MuRF1 Am J Physiol Endocrinol Metab 2004 287 E591 E601 10.1152/ajpendo.00073.2004 15100091

[B7] Banik NL Matzelle D Terry E Hogan EL A new mechanism of methylprednisolone and other corticosteroids action demonstrated in vitro: inhibition of a proteinase (calpain) prevents myelin and cytoskeletal protein degradation Brain Res 1997 748 205 210 10.1016/S0006-8993(96)01302-9 9067463

[B8] Wang M Shen F Shi LH Xi T Li XF Chen X Protective effect of prednisolone on ischemia-induced liver injury in rats World J Gastroenterol 2008 14 4332 4337 10.3748/wjg.14.4332 18666321 PMC2731184

[B9] Maes K Testelmans D Cadot P Deruisseau K Powers SK Decramer M Effects of acute administration of corticosteroids during mechanical ventilation on rat diaphragm Am J Respir Crit Care Med 2008 178 1219 1226 10.1164/rccm.200702-296OC 18849500 PMC3266049

[B10] Pearl JM Plank DM McLean KM Wagner CJ Duffy JY Glucocorticoids Improve Calcium Cycling in Cardiac Myocytes after Cardiopulmonary Bypass J Surg Res 2009 19726057 10.1016/j.jss.2009.05.001 PMC2888998

[B11] Glanemann M Strenziok R Kuntze R Munchow S Dikopoulos N Lippek F Ischemic preconditioning and methylprednisolone both equally reduce hepatic ischemia/reperfusion injury Surgery 2004 135 203 214 10.1016/j.surg.2003.08.011 14739856

[B12] Pearl JM Nelson DP Schwartz SM Wagner CJ Bauer SM Setser EA Glucocorticoids reduce ischemia-reperfusion-induced myocardial apoptosis in immature hearts Ann Thorac Surg 2002 74 830 836 10.1016/S0003-4975(02)03843-2 12238847

[B13] Wang H Wu YB Du XH Effect of dexamethasone on nitric oxide synthase and Caspase-3 gene expressions in endotoxemia in neonate rat brain Biomed Environ Sci 2005 18 181 186 16131021

[B14] Wang M Sakon M Umeshita K Okuyama M Shiozaki K Nagano H Prednisolone suppresses ischemia-reperfusion injury of the rat liver by reducing cytokine production and calpain mu activation J Hepatol 2001 34 278 283 10.1016/S0168-8278(00)00017-9 11281557

[B15] DeRuisseau KC Shanely RA Akunuri N Hamilton MT Van Gammeren D Zergeroglu AM Diaphragm unloading via controlled mechanical ventilation alters the gene expression profile Am J Respir Crit Care Med 2005 172 1267 1275 10.1164/rccm.200503-403OC 16126937 PMC2718415

[B16] DeRuisseau KC Kavazis AN Deering MA Falk DJ Van Gammeren D Yimlamai T Mechanical ventilation induces alterations of the ubiquitin-proteasome pathway in the diaphragm J Appl Physiol 2005 98 1314 1321 10.1152/japplphysiol.00993.2004 15557010

[B17] Shanely RA Zergeroglu MA Lennon SL Sugiura T Yimlamai T Enns D Mechanical ventilation-induced diaphragmatic atrophy is associated with oxidative injury and increased proteolytic activity Am J Respir Crit Care Med 2002 166 1369 1374 10.1164/rccm.200202-088OC 12421745

[B18] Goll DE Thompson VF Li H Wei W Cong J The calpain system Physiol Rev 2003 83 731 801 12843408 10.1152/physrev.00029.2002

[B19] Reagan-Shaw S Nihal M Ahmad N Dose translation from animal to human studies revisited FASEB J 2008 22 659 661 10.1096/fj.07-9574LSF 17942826

[B20] Dekhuijzen PN Gayan-Ramirez G de Bock V Dom R Decramer M Triamcinolone and prednisolone affect contractile properties and histopathology of rat diaphragm differently J Clin Invest 1993 92 1534 1542 10.1172/JCI116732 8376603 PMC288300

[B21] Betters JL Criswell DS Shanely RA Van Gammeren D Falk D DeRuisseau KC Trolox attenuates mechanical ventilation-induced diaphragmatic dysfunction and proteolysis Am J Respir Crit Care Med 2004 170 1179 1184 10.1164/rccm.200407-939OC 15374845

[B22] Auclair D Garrel DR Chaouki ZA Ferland LH Activation of the ubiquitin pathway in rat skeletal muscle by catabolic doses of glucocorticoids Am J Physiol 1997 272 C1007 C1016 9124503 10.1152/ajpcell.1997.272.3.C1007

[B23] Dardevet D Sornet C Taillandier D Savary I Attaix D Grizard J Sensitivity and protein turnover response to glucocorticoids are different in skeletal muscle from adult and old rats. Lack of regulation of the ubiquitin-proteasome proteolytic pathway in aging J Clin Invest 1995 96 2113 2119 10.1172/JCI118264 7593595 PMC185859

[B24] Deval C Mordier S Obled C Bechet D Combaret L Attaix D Identification of cathepsin L as a differentially expressed message associated with skeletal muscle wasting Biochem J 2001 360 143 150 10.1042/0264-6021:3600143 11696001 PMC1222211

[B25] Sohar I Nagy I Heiner L Kovacs Z Guba F Proteases and proteinase inhibitors in experimental glucocorticosteroid myopathy Acta Physiol Acad Sci Hung 1982 60 43 51 6764081

[B26] Zhao W Pan J Zhao Z Wu Y Bauman WA Cardozo CP Testosterone protects against dexamethasone-induced muscle atrophy, protein degradation and MAFbx upregulation J Steroid Biochem Mol Biol 2008 110 125 129 10.1016/j.jsbmb.2008.03.024 18436443

[B27] Rifai Z Welle S Moxley RT III Lorenson M Griggs RC Effect of prednisone on protein metabolism in Duchenne dystrophy Am J Physiol 1995 268 E67 E74 7840185 10.1152/ajpendo.1995.268.1.E67

[B28] Alderton JM Steinhardt RA Calcium influx through calcium leak channels is responsible for the elevated levels of calcium-dependent proteolysis in dystrophic myotubes J Biol Chem 2000 275 9452 9460 10.1074/jbc.275.13.9452 10734092

[B29] Sur P Sribnick EA Patel SJ Ray SK Banik NL Dexamethasone decreases temozolomide-induced apoptosis in human gliobastoma T98G cells Glia 2005 50 160 167 10.1002/glia.20168 15685605

[B30] Ostwald K Hayashi M Nakamura M Kawashima S Subcellular distribution of calpain and calpastatin immunoreactivity and fodrin proteolysis in rabbit hippocampus after hypoxia and glucocorticoid treatment J Neurochem 1994 63 1069 1076 10.1046/j.1471-4159.1994.63031069.x 8051548

[B31] Schwartz SM Duffy JY Pearl JM Goins S Wagner CJ Nelson DP Glucocorticoids preserve calpastatin and troponin I during cardiopulmonary bypass in immature pigs Pediatr Res 2003 54 91 97 10.1203/01.PDR.0000065730.79610.7D 12646718

[B32] Niazi Z Flodin P Joyce L Smith J Mauer H Lillehei RC Effects of glucocorticosteroids in patients undergoing coronary artery bypass surgery Chest 1979 76 262 268 10.1378/chest.76.3.262 380941

[B33] Schroeder VA Pearl JM Schwartz SM Shanley TP Manning PB Nelson DP Combined steroid treatment for congenital heart surgery improves oxygen delivery and reduces postbypass inflammatory mediator expression Circulation 2003 107 2823 2828 10.1161/01.CIR.0000070955.55636.25 12756159

[B34] Toledo-Pereyra LH Lin CY Kundler H Replogle RL Steroids in heart surgery: a clinical double-blind and randomized study Am Surg 1980 46 155 160 7377659

[B35] Leijendekker WJ Passaquin AC Metzinger L Ruegg UT Regulation of cytosolic calcium in skeletal muscle cells of the mdx mouse under conditions of stress Br J Pharmacol 1996 118 611 616 8762085 10.1111/j.1476-5381.1996.tb15445.x PMC1909736

[B36] Maes K Testelmans D Powers S Decramer M Gayan-Ramirez G Leupeptin Inhibits Ventilator-induced Diaphragm Dysfunction in Rats Am J Respir Crit Care Med 2007 175 1134 1138 10.1164/rccm.200609-1342OC 17379854

[B37] Sassoon CS Zhu E Pham HT Nelson RS Fang L Baker MJ Acute effects of high-dose methylprednisolone on diaphragm muscle function Muscle Nerve 2008 38 1161 1172 10.1002/mus.21048 18671291

[B38] Wang L Luo GJ Wang JJ Hasselgren PO Dexamethasone stimulates proteasome- and calcium-dependent proteolysis in cultured L6 myotubes Shock 1998 10 298 306 10.1097/00024382-199810000-00011 9788663

[B39] Yin HN Chai JK Yu YM Shen CA Wu YQ Yao YM Regulation of signaling pathways downstream of IGF-I/insulin by androgen in skeletal muscle of glucocorticoid-treated rats J Trauma 2009 66 1083 1090 10.1097/TA.0b013e31817e7420 19359918 PMC4085982

[B40] Beyette J Mason GG Murray RZ Cohen GM Rivett AJ Proteasome activities decrease during dexamethasone-induced apoptosis of thymocytes Biochem J 1998 332 Pt 2 315 320 9601058 10.1042/bj3320315 PMC1219484

